# Breeding histories and selection criteria for oilseed rape in Europe and China identified by genome wide pedigree dissection

**DOI:** 10.1038/s41598-017-02188-z

**Published:** 2017-05-15

**Authors:** Xiaohua Wang, Yan Long, Nian Wang, Jun Zou, Guangda Ding, Martin R. Broadley, Philip J. White, Pan Yuan, Qianwen Zhang, Ziliang Luo, Peifa Liu, Hua Zhao, Ying Zhang, Hongmei Cai, Graham J. King, Fangsen Xu, Jinling Meng, Lei Shi

**Affiliations:** 1 National Key Laboratory of Crop Genetic Improvement, Huazhong Agricultural University, Wuhan, 430070 China; 2 Key Lab of Cultivated Land Conservation, Ministry of Agriculture, Microelement Research Centre, Huazhong Agricultural University, Wuhan, 430070 China; 3 Biotechnology Research Institute, Chinese Academy of agricultural Science, Beijing, 100081 China; 4 College of Horticulture & Forestry Sciences, Huazhong Agricultural University, Wuhan, 430070 China; 5 Plant and Crop Sciences Division, School of Biosciences, University of Nottingham, Sutton Bonington Campus, Loughborough, LE12 5RD United Kingdom; 6 The James Hutton Institute, Invergowrie, Dundee, DD2 5DA United Kingdom; 7 King Saud University, Riyadh, 11451 Saudi Arabia; 8 Southern Cross Plant Science, Southern Cross University, Lismore, NSW 2480 Australia

## Abstract

Selection breeding has played a key role in the improvement of seed yield and quality in oilseed rape (*Brassica napus* L.). We genotyped Tapidor (European), Ningyou7 (Chinese) and their progenitors with the *Brassica* 60 K Illumina Infinium SNP array and mapped a total of 29,347 SNP markers onto the reference genome of Darmor-*bzh*. Identity by descent (IBD) refers to a haplotype segment of a chromosome inherited from a shared common ancestor. IBDs identified on the C subgenome were larger than those on the A subgenome within both the Tapidor and Ningyou7 pedigrees. IBD number and length were greater in the Ningyou7 pedigree than in the Tapidor pedigree. Seventy nine QTLs for flowering time, seed quality and root morphology traits were identified in the IBDs of Tapidor and Ningyou7. Many more candidate genes had been selected within the Ningyou7 pedigree than within the Tapidor pedigree. These results highlight differences in the transfer of favorable gene clusters controlling key traits during selection breeding in Europe and China.

## Introduction

Oilseed rape (OSR, canola, *Brassica napus* L.) is the third largest source of vegetable oil in the world^1^, providing food, feed and fuel. In recent years, the demand for OSR has increased due to the rising demand for biofuels^2^. Over 70 million tonnes of rapeseed are produced annually across the world (FAOSTAT)^3^.

**Figure 1:**
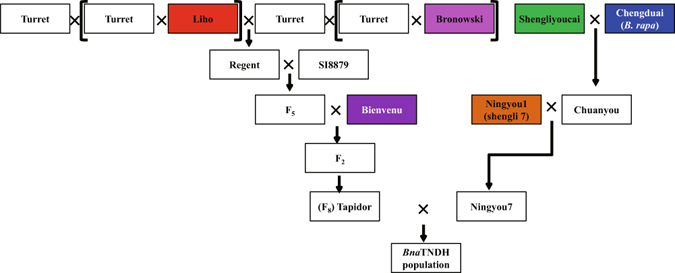
Schematic of genetic transfer in the pedigrees of cultivars Tapidor and Ningyou7. Cultivars Regent, Bienvenu, Liho and Bronowski are ancestors of cultivar Tapidor, with all cultivars developed in European countries or Canada. Cultivars Ningyou1, Chuanyou2, Shengliyoucai and Chengduai are ancestors of cultivar Ningyou7, which were all bred in Asian countries and most in China. Of these, cultivar Chengduai is *Brassica rapa* (A genome), and the remainder are *Brassica napus* (AC genome). The *Bna*TNDH population is derived from a cross between homozygous lines derived from cultivars Tapidor and Ningyou7.

*Brassica napus* (AC genome) is a domesticated allotetraploid, arising from natural hybridization of the diploid species *B. rapa* (A) and *B. oleracea* (C) over 7500 years ago^4^. Modern canola-type OSR arose from the selection of improved seed composition attributes following introduction of beneficial alleles from exotic germplasm. The pedigrees of the cultivars Tapidor and Ningyou7 are well-documented^5–10^. Tapidor was derived from a cross between Regent and Bienvenu^5^. It has a high yield and oil content, low erucic acid and glucosinolate content in the seed, and was one of the most popular modern cultivars in the 1990s in northern Europe^5, 6^ (Fig. 1). Regent was selected from the offspring of a cross between Liho and Bronowski. Liho is a silage *B. napus* cultivar with low seed erucic acid content^6, 7^. Bronowski is a cultivar with low seed glucosinolate content^6^.

The first popular *B. napus* variety in China was introduced from Japan in the 1930s and renamed as Shengliyoucai (Victory OSR) in the 1950s. Shengliyoucai was crossed with Chengduai, an early flowering *B. rapa* variety widely planted in the Sichuan province of China, and Chuanyou2 selected from the resulting progeny^8, 9^. Ningyou1, a semi-winter cultivar with early flowering time and good yield was crossed with Chuanyou2, and Ningyou7 resulted from their offspring^10^. Ningyou7 had high seed oil content, earlier flowering time and greater seed yield than its ancestors^6^.

In *B. napus* most traits affecting yield and quality have a complex polygenic basis^11–14^. Mapping of quantitative trait loci (QTL), which is used to identify genomic regions that are responsible for trait variation, is based on associations between polymorphic markers and phenotypic values^13, 14^ in unselected biparental segregating populations. This method can reveal the genetic basis of complex traits for which prior knowledge may be limited and the lack of which would otherwise present a major constraint to crop breeding^15^. The *Bna*TNDH mapping population was generated through anther culture of the F_1_ generation of a cross between Tapidor and Ningyou7^16^. Within this population, five to 18 QTLs for flowering time have been detected in 12 winter microenvironments and three spring microenvironments, with about 60% of the phenotypic variation for flowering time attributed to genetic effects^17^. A QTL on linkage group A10, *qFT10-4*, was detected in a spring-cropped environment. BnaA.FLC*.10* has been identified as a candidate gene that has been shown to be an important determinant of winter versus spring type OSR^17^. In another study with the same population grown in ten environments across three geographic regions^18^, 437 SNPs associated with flowering time were detected, some of which were associated with known genes such as orthologs of *APETALA1* in *A. thaliana*. Genetic loci contributing to seed erucic acid content^11–14, 16, 19^, seed and leaf glucosinolate content^14, 20, 21^, and root traits affecting phosphorus (Pi) acquisition^22, 23^ have also been identified in *B. napus* by QTL mapping or through genome wide associated studies (GWAS).

Identity by descent (IBD) refers to a haplotype segment of a chromosome inherited from a shared common ancestor^24^. IBD segments have been employed to determine the origin of low diversity regions in humans to detect rare alleles related to diastolic blood pressure^25^. Selection tends to increase the number of IBD segments among individuals in a population. Strong and very recent selection in the human genome has been identified by scanning regions with excess IBD sharing^26, 27^.

Based on the differences between the number of segregating sites and the average number of nucleotide differences, Tajima’s D test can be used to distinguish a DNA sequence diverging randomly from another that is diverging due to a non-random process, including directional selection or balancing selection, demographic expansion or contraction, genetic hitchhiking or introgression^28^. In maize, Tajima’s D test has been used to establish the genome location(s) where selection has occurred^29^. When compared with long term selection in natural environments, domestication promotes rapid phenotypic evolution through artificial selection^30^. Random genetic drift is a powerful mechanism that enables populations of finite size to acquire genetic structure^31^. This can be seen in the domestication bottleneck and associated reduction in genetic diversity for specific target genes subject to artificial selection, with the magnitude and variance of this reduction across loci providing insights into the demographic history of domestication^32^.

In this study, homozygous lines derived from Tapidor, Ningyou7 and their ancestors were screened using the genome-anchored *Brassica* 60 K Illumina Infinium SNP array. The objectives were (1) to survey IBD regions in the pedigree of these cultivars to identify differences in the extent of IBD transfer from their ancestors to offspring; (2) to identify candidate genes underlying QTL within IBD regions for flowering time, seed quality and root morphology traits and uncover any gene clusters transferred from the progenitors to Tapidor and Ningyou7, (3) to identify the genome regions where breeding selection has occurred using Tajima’s D and gene diversity tests.

## Results

### SNP distribution in the Tapidor and Ningyou7 pedigrees and alignment to the *B. napus* reference genome

**Figure 2:**
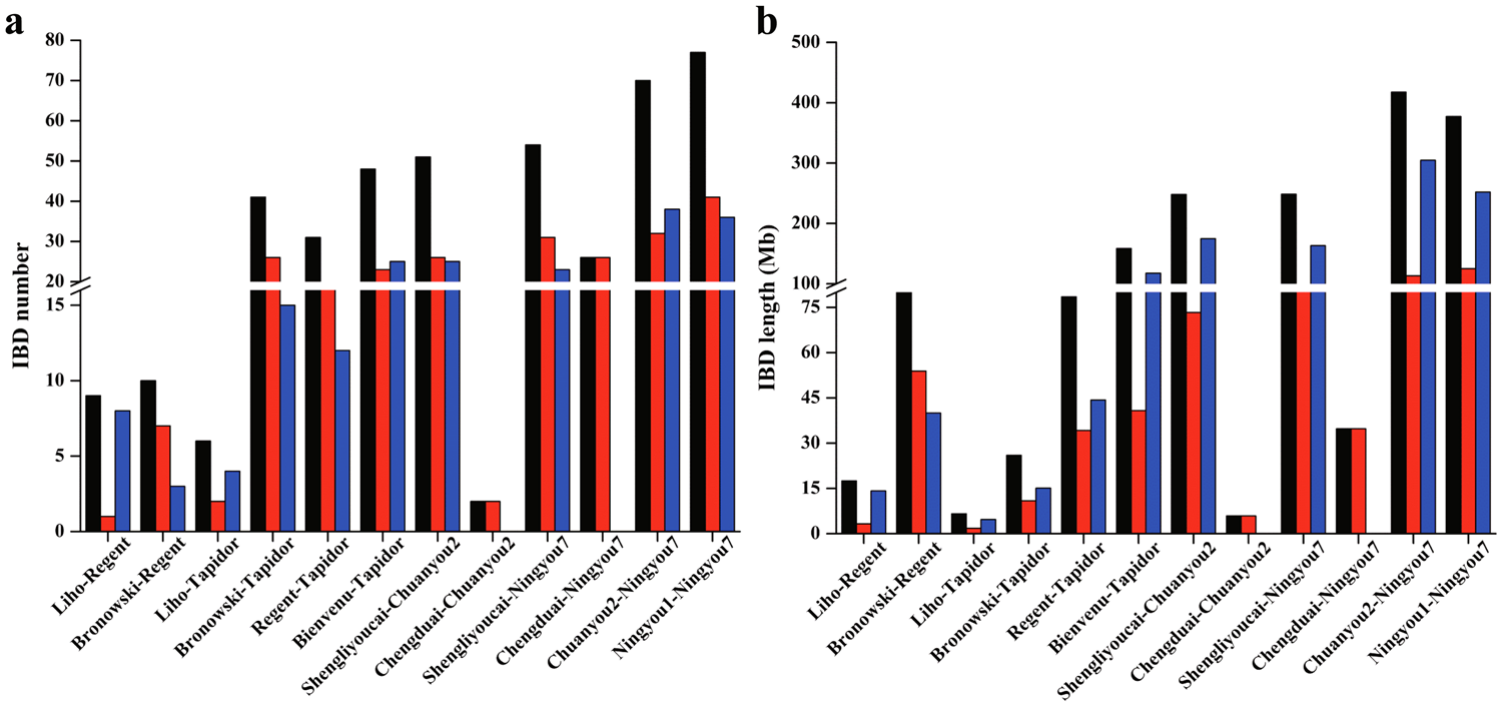
The number and length of identity by descent (IBD) blocks revealing the transfer of haplotype segments in the Tapidor and Ningyou7 pedigree cultivars. Number (**a**) and length (**b**) of IBD in the A and C sub-genomes of cultivars in the Tapidor and Ningyou7 pedigrees. The transfer of IBD from each progenitor to cultivars Regent, Tapidor, Chuanyou2 or Ningyou7 in the whole genome (black), A subgenome (red) and C subgenome (blue) are indicated.

Tapidor, Ningyou7 and their eight ancestors (inbred selections) were screened using the 60 K *Brassica* Infinium SNP array, and a total of 29,347 high–quality SNP markers were mapped to 643.7 Mb across the 19 chromosomes of the 849.7 Mb reference genome of *B. napus* cultivar Darmor-*bzh* (Supplementary Table S1). C03 was the longest chromosome, represented by ~60.5 Mb in the Tapidor and Ningyou7 pedigrees and ~60.6 Mb in cultivar Darmor-*bzh*, while A10 was the shortest chromosome, with ~17.4 Mb in both the Tapidor and Ningyou7 pedigrees as well as Darmor-*bzh* (Fig. 2 **)**. For all 19 chromosomes, the lengths represented within the Tapidor and Ningyou7 pedigrees were slightly shorter than those of Darmor-*bzh*. The total length of the A subgenome (A01 to A10) was 238.8 Mb and of the C subgenome (C01 to C09) 404.9 Mb (Supplementary Table S1). An average density of 46 SNP per Mb was recorded in the genomes of the Tapidor and Ningyou7 pedigrees. Compared with other chromosomes, SNP marker densities were lower in A02 (30 SNP per Mb), C05 (20) and C09 (22) (Supplementary Table S1).

### Difference in IBD transfer between Tapidor and Ningyou7 pedigrees

Identity by descent (IBD) detected by pairwise comparison of cultivars within both pedigrees was calculated, along with the number and length of IBD blocks in the A and C subgenomes (Fig. 2, and Supplementary Table S2), and for both measures found to be greater in the Ningyou7 pedigree. Within the A subgenome of Tapidor pedigree, 78 IBD blocks with a total length of 161.2 Mb were identified, and for the C subgenome 67 IBD blocks (304.5 Mb). Within the Ningyou7 pedigree, 158 IBD blocks (438 Mb) were identified in the A subgenome and 122 IBD blocks (894.2 Mb) in the C subgenome (Fig. 2).

Most pairwise comparisons of cultivars detected more IBD blocks in the A subgenome than in the C subgenome, e.g. Bronowski *vs* Tapidor, Regent *vs* Tapidor, Bienvenu *vs* Tapidor, Shengliyoucai *vs* Ningyou7 and Ningyou1 *vs* Ningyou7 (Fig. 2). In contrast, there were fewer IBD blocks in the A subgenome for Liho *vs* Regent, Liho *vs* Tapidor and Ningyou7 *vs* Chuanyou2 (Fig. 2). No significant difference between subgenomes was observed for Bronowski *vs* Regent and Shengliyoucai *vs* Chuanyou2 (Fig. 2). The total lengths of the IBD blocks were shorter in the A subgenome than in the C subgenome for all pairwise comparisons of cultivars apart from Bronowski *vs* Tapidor. Overall the A subgenome showed more genetic variation than the C subgenome.

Within the Tapidor pedigree, the progenitor cultivar comparison of Bronowski *vs* Regent had the most (48) and longest (158.4 Mb) IBD blocks, whilst Liho *vs* Tapidor had the least (6), and shortest (6.6 Mb) IBD blocks (Fig. 2). Within the Ningyou7 pedigree, Ningyou1 *vs* Ningyou7 had the most (77), and Chengduai *vs* Chuanyou2 the least (2) and shortest (5.9 Mb) IBD blocks, while Chunayou2 *vs* Ningyou7 had the longest (417.7 Mb) IBD blocks (Fig. 2 and Supplementary Table S2). These results suggest that the IBD block length decreases with increasing number of generations and, hence, recombination cycles.

**Figure 3:**
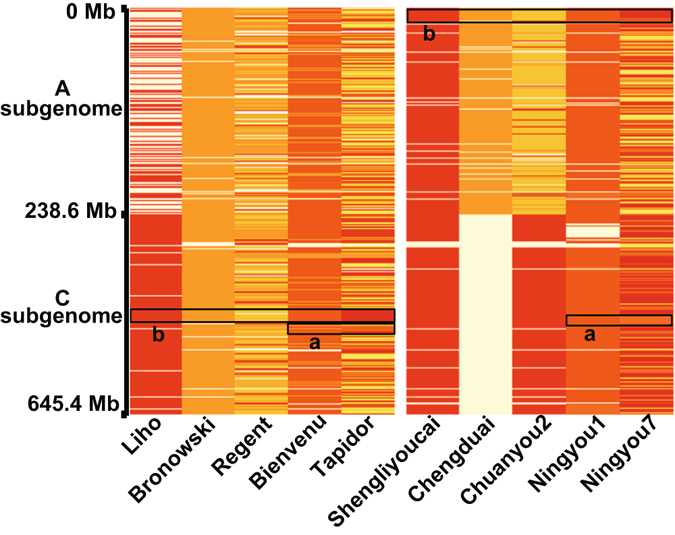
The transfer of identity by descent (IBD) blocks from earlier cultivars to the Tapidor and Ningyou7 cultivars based on SNP markers. Progenitor genomes are indicated by shading for Tapidor (left side) and for Ningyou7 (right). The breeding of both Tapidor and Ningyou7 has selected for IBD from both recent (**a**) and distant (**b**) ancestor cultivars.

Two paths of haplotype transfer are possible within the Tapidor and Ningyou7 pedigrees, either via direct linear transfer from original parent to progeny in recent breeding crosses, or indirectly by transfer from intermediate parents generated in earlier breeding crosses (Fig. 3). Of the 79 directly transferred IBD blocks (corresponding to 236.9 Mb) identified within the Tapidor pedigree, 31 (78.5 Mb) were derived from Regent and 48 (158.4 Mb) from Bienvenu, with 47 indirectly transferred IBD blocks, including six (6.6 Mb) originating from Liho and 41 (26.0 Mb) from Bronowski (Table 1 and Fig. 3b). In contrast, of the 147 directly transferred IBD blocks identified in the Ningyou7 pedigree, 70 (417.7 Mb) were derived from Chuangyou2 and 77 (377.2 Mb) from Ningyou1, with 80 indirectly transferred IBDs, including 54 (248.5 Mb) from Shengliyoucai and 26 (34.8 Mb) from Chengduai (*B. rapa*) (Table 1 and Fig. 3a).

**Table 1:** Number and total size of identity by descent (IBD) blocks by direct and indirect genetic transfer in the breeding of Tapidor and Ningyou7.

Pedigree	Group	Size (Mb)	Number of IBD	Path of genetic transfer	Total IBD size(Mb)
Tapidor	Liho *vs* Tapidor	6.6	6	indirect	32.6
	Bronowski *vs* Tapidor	26.0	41	indirect	
	Regent *vs* Tapidor	78.5	31	direct	236.9
	Bienvenu *vs* Tapidor	158.4	48	direct	
	Shengliyoucai *vs* Ningyou7	248.5	54	indirect	283.3
Ningyou7	Chengduai *vs* Ningyou7	34.8	26	indirect	
	Chuanyou2 *vs* Ningyou7	417.7	70	direct	794.9
	Ningyou1 *vs* Ningyou7	377.2	77	direct	

**Figure 4:**
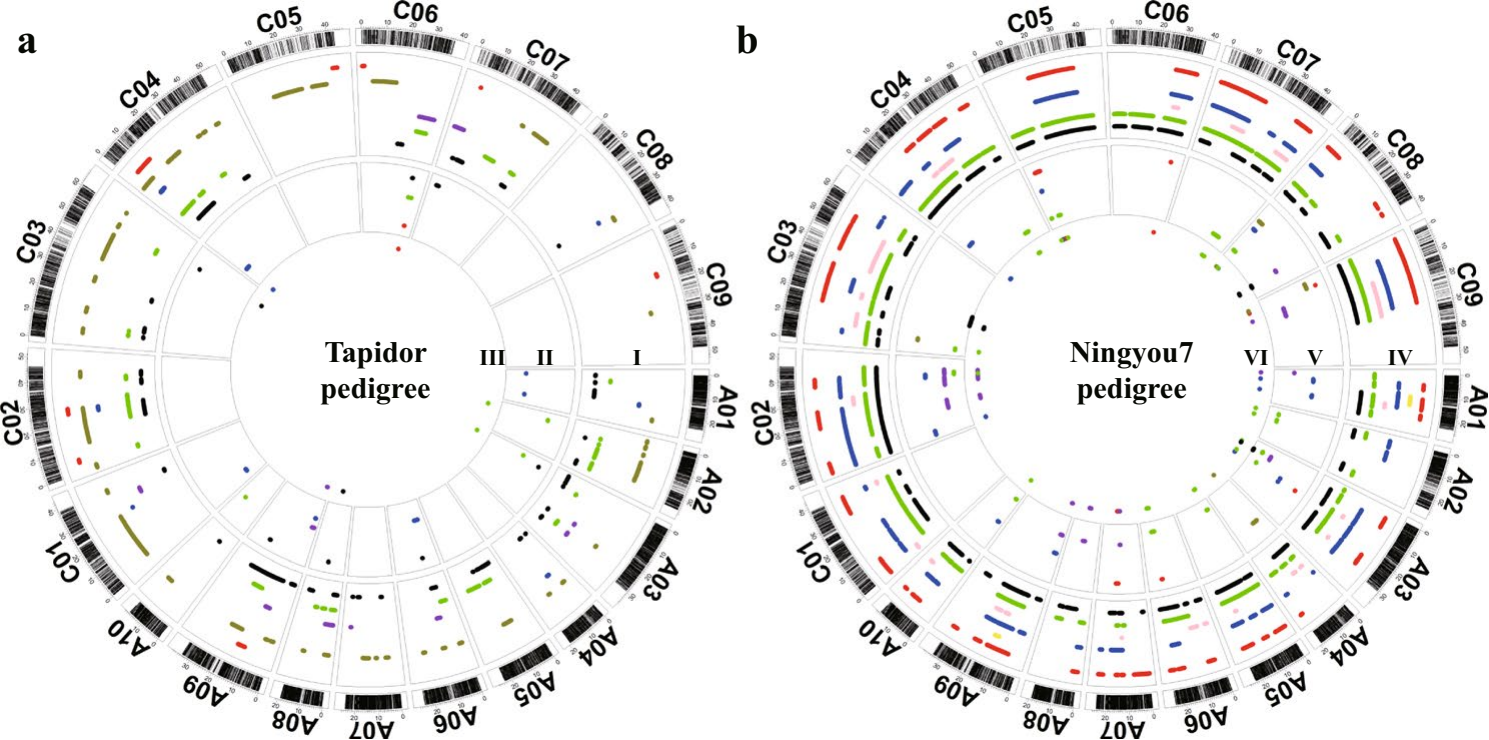
Distribution of QTLs detected for flowering time, seed quality and root morphology traits within the *Bna*TNDH population, and the candidate genes underlying the QTL region located in IBDs of cultivars of the Tapidor and Ningyou7 pedigrees. The outermost circle represents the SNP markers located on the genome of cultivar Darmor-*bzh*. Circle ‘I’ indicates the locations of IBD blocks on each of the chromosomes within the Tapdior pedigree; ‘IV’ within the Ningyou7 pedigree. Circle ‘II’ represents the additive effect QTLs for different agronomic traits located in the IBD regions within the Tapidor pedigree, and ‘V’ within the Ningyou7 pedigree. Circle ‘III’ demonstrates the candidate genes located in the QTL regions within the Tapidor pedigree and ‘VI’ within the Ningyou7 pedigree.

The physical positions corresponding to QTLs identified for flowering time, seed oil content, seed erucic acid content, seed glucosinolate content and root morphology traits were mapped on the genome of the Tapidor and Ningyou7 pedigrees (Luo *et al*. unpublished data). A notable number of consensus QTL and genes were identified in the IBD block regions. These included those for flowering time (20 of 68 consensus QTL and 37 genes), seed oil content (21 of 73 QTL, 42 genes), seed glucosinolate content (15 of 45 QTL, 40 genes), and seed erucic acid content (7 of 35 QTL, 17 genes), as well as for root traits (16 of 56 QTL, 42 genes) (Fig. 4 and Supplementary Table S2).

### Evidence for gene level breeding selection in the Tapidor and Ningyou7 pedigrees

**Figure 5:**
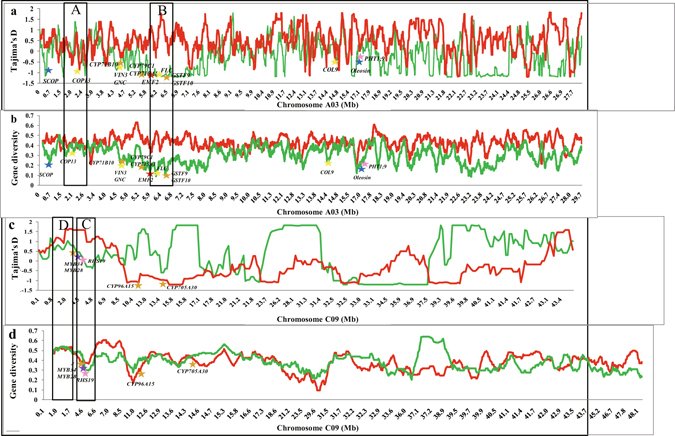
Variation in Tajima’s D (**a,c**) and gene diversity (**b,d**) values across chromosome A03 (**a,b**) and chromosome C09 (**c,d**) for cultivars in the Tapidor (red) and Ningyou7 (green) pedigrees. Key genes controlling flowering time (yellow stars), seed oil content (blue stars), seed glucosinolate content (orange star), seed erucic acid content (red stars), seed protein content (purple stars), and root morphological traits (pink stars) are indicated. Examples of genes in regions with Tajima’s D <0 and gene diversity >0.3, implying early selection during the breeding process (Box A), in regions with Tajima’s D <0 and gene diversity <0.3, implying later selection in the breeding process (Box B), in regions with Tajima’s D >0 and gene diversity <0.3, indicating selection before the pedigrees shown here (Box C), and in regions with Tajima’s D >0 and gene diversity >0.3, implying no selection on the locus (Box D), are illustrated.

Based on the neutral selection theory for populations^28^, Tajima’s D values were calculated in each of the Tapidor and Ningyou7 pedigrees. Tajima’s D test allows detection of the location of selection at the DNA level. A value of 0.44 across the genome was associated with the Tapidor pedigree, compared with only 0.02 in the Ningyou7 pedigree. The value ranged from −0.23 (C09) to 0.94 (C01) in the Tapidor pedigree, and from −0.40 (C02) to 0.48 (C05) in the Ningyou7 pedigree (Supplementary Table S2). Within the Tapidor pedigree negative values were associated with C09, while in the Ningyou7 pedigree they were associated with two A subgenome chromosomes (A03, A05) and six C subgenome chromosomes (C02, C03, C04, C06, C07 and C09), indicating that directional selection had increased the rare alleles (Fig. 5, Supplementary Fig. S1 and Supplementary Table S2). We identified four classes of Tajima D values accounting for the total SNP variation within the Tapidor (T_pedigree_) and Ningyou7 pedigrees (N_pedigree_), with Class 1 having (−2 to −1): 5% of T_pedigree_ vs 13% N_pedigree_; Class 2 (−1 to 0): 20% of T_pedigree_ vs 34% N_pedigree_; Class 3 (0 to 1): T_pedigree_ 48% vs 40% N_pedigree_; Class 4 (1 to 2): T_pedigree_ 13% vs 13% N_pedigree_ (Supplementary Fig. S1).

Gene diversity across all chromosomes was similar for both pedigrees (Supplementary Table S2). In the Tapidor pedigree, mean values ranged from 0.35 (C05) to 0.44 (A04) with a mean of 0.40, compared with 0.3 (A02) to 0.52 (C01) with a mean of 0.36 in the Ningyou7 pedigree. The mean polymorphism information content (PIC) was slightly greater in the Tapidor pedigree (0.34, ranging from 0.28 in C05 to 0.38 in A04) than in the Ningyou7 pedigree (0.31, ranging from 0.24 in A02 to 0.45 in C01) (Supplementary Fig. S2 and Supplementary Table S2).

**Figure 6:**
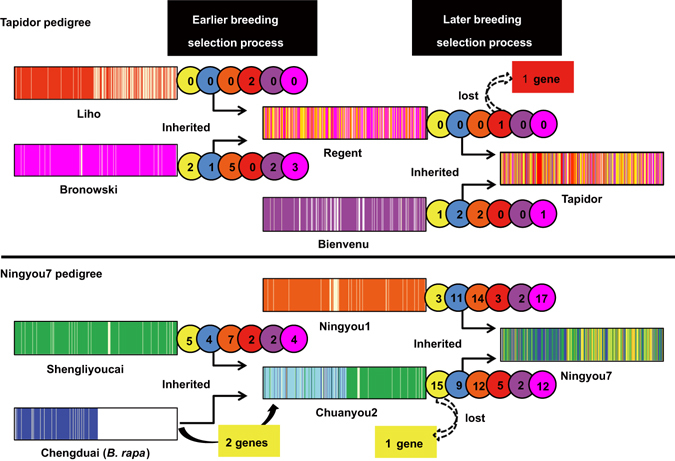
The transfer of candidate genes underlying the QTL region located in IBDs from earlier cultivars to the Tapidor and Ningyou7 cultivars in the breeding process based on SNP markers. Genomes are represented as rectangular boxes and the chromosomes of progenitor cultivars are indicated in different colors. Colored discs represent the number of key genes originating from each ancestor cultivar that have contributed to: flowering time (yellow), seed oil content (blue), seed glucosinolate content (orange), seed erucic acid content (red), seed protein content (purple), and root morphological traits (pink). Rectangular boxes represent genes that were not transferred to Tapidor and Ningyou7 for flowering time (yellow) and seed erucic acid content (red). The number in parenthesis is the theoretical genetic information transferred from progenitor cultivars to Tapidor and Ningyou7.

Two primary artificial selection processes are apparent in the breeding history of Tapidor and Ningyou7 (Fig. 5). Where a locus has a Tajima’s D <0 and gene diversity <0.3 (Fig. 5A), the artificial breeding selection of the locus is more likely to have occurred during the earlier breeding cycles. Where a locus has a Tajima’s D <0 and gene diversity >0.3 (Fig. 5B), the artificial breeding selection of the locus is likely to have occurred in a later round of breeding selection. Where a locus has a Tajima’s D >0 and gene diversity <0.3 (Fig. 5C), the artificial breeding selection of the locus is more likely to have taken place prior to the founding cultivars. Finally, where a locus has a Tajima’s D >0 and gene diversity >0.3 (Fig. 5D), there is no strong evidence for artificial breeding selection occurring at the locus (Figs 5 and 6)^30^. A total of 42 genes, 15 within the Tapidor and 27 within the Ningyou7 pedigrees, were detected where genetic transfer was associated within the earlier breeding selection process (Fig. 6 and Supplementary Table S2). Among these genes, two for flowering time and one for seed erucic acid content in the Tapidor pedigree appeared to have been subject to breeding selection. In the Ningyou7 pedigree, the genes involved in breeding selection included five for flowering time, two for seed erucic acid content, three for seed glucosinolate content and two for root morphology and phosphorus uptake. In the later breeding selection process, only two genes in Tapidor, but 116 in Ningyou7 were detected as being associated with genetic transfer (Fig. 6 and Supplementary Table S2). Of these, only one from the Tapidor pedigree and 65 from the Ningyou7 pedigree appeared to have been subject to breeding selection. The sole gene detected within the Tapidor pedigree, BnaA.FAE*1*, is an ortholog of *KCS18* (3-ketoacyl-CoA synthase 18) located on A08, and represents one of the key domestication genes introduced from Liho, and known to contribute to modern ‘double-low’ canola, by reducing seed erucic acid content. The genes within the Ningyou7 pedigree included 15 for flowering time, ten for oil content, 18 for glucosinolate content, ten for erucic acid content, two for seed protein content, and ten for root morphology and phosphorus uptake (Fig. 6 and Supplementary Table S2).

### Favorable gene clusters transferred in the breeding of Tapidor and Ningyou7

**Figure 7:**
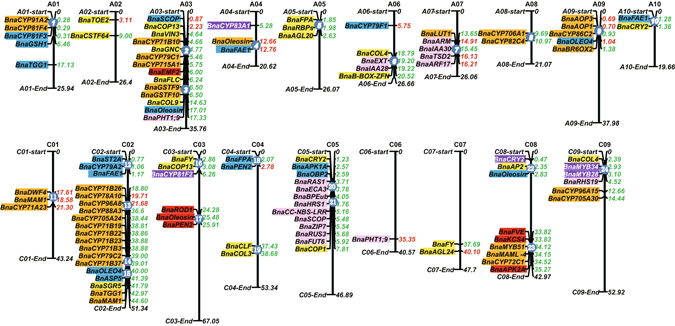
The distribution of gene clusters controlling important agronomic traits located on the physical map of *B. napus*. Key genes controlling flowering time (yellow boxes), seed oil content (blue boxes), seed glucosinolate content (orange boxes), seed erucic acid content (red boxes), seed protein content (purple boxes), and root morphological traits (pink boxes) detected within QTL regions in the *Bna*TNDH mapping population plotted on the 19 chromosomes of Darmor-*bzh*. Numbers to the right of each chromosome indicate the position of beneficial alleles from the Tapidor pedigree (red) or the Ningyou7 pedigree (green). The blue disc with white numbers on chromosomes indicates the gene clusters of important agronomic traits in the two cultivar pedigrees.

Genetic hitchhiking (linkage drag) effects contribute to linkage disequilibrium (LD)^33^. The LD of the ten cultivars within the Tapidor and Ningyou7 pedigrees was calculated and used to estimate the extent of linkage drag. A mean correlation coefficient (r^2^) of 0.227 in the pedigree lineage indicated that a significant genetic relationship might exist at the whole genome level. When r^2^ = 0.2, the LD decay was 0.7 Mb. Thus, where genes are located within a region of LD ≤0.7 Mb in the *B. napus* genome they will form a cluster, and are more likely to be transferred as an intact haplotype block from the parents to offspring. A total of 24 favorable gene clusters, 10 in the A subgenome and 14 in the C subgenome were identified. These were associated with seed quality, flowering time, and root morphology traits linked to phosphorus uptake. They were located on all chromosomes apart from A02, C06 and C07 (Fig. 7 and Supplementary Table S2). Genetic transfer was detected within the Ningyou7 pedigree (18 clusters), the Tapidor pedigree (2 clusters) and within both pedigrees (4 clusters). In addition, 12 gene clusters were associated with combinations of two or more important agronomic traits, such as flowering time, root morphology and phosphorus uptake (No 6, A06), or flowering time and seed oil content (No. 14, C02; No. 20, C05; No. 22, C08) (Supplementary Table S2).

## Discussion

Tapidor is a double-low (low seed glucosinolate and low seed erucic acid content) winter *B. napus* cultivar developed in Europe. Ningyou7 is a double-high (high seed glucosinolate and high seed erucic acid content) semi-winter *B. napus* cultivar developed in China. The flowering time of Tapidor is later than that of Ningyou7^17^, and has a longer primary root and fewer lateral roots than Ningyou7^23^. Agronomic and seed traits within the pedigrees of both these cultivars have undergone substantial improvement through extensive breeding efforts. These cultivars were crossed to generate the *Bna*TNDH biparental doubled haploid segregating population, which has been used extensively to understand the genetic basis of many agronomic and economically important traits in *B. napus* ^1, 13, 14, 16, 17, 19, 23, 33–36^. Uncovering the associated underlying patterns of allelic variation in the context of the sequenced genome is anticipated to provide valuable knowledge that will guide subsequent advances in breeding^1, 11^.

In this study, Tapidor, Ningyou7 and their eight progenitor ancestors were screened with the 60 K *Brassica* Infinium SNP array. A total of 29,347 high–quality SNP markers were mapped to the 19 pseudomolecule chromosomes of Darmor-*bzh* (Supplementary Table S1). Using the SNP markers of Tapidor and Ningyou7 as the reference for each pedigree, IBD regions detected from pairwise comparisons of cultivars in each of the pedigrees were assessed (Fig. 4 and Supplementary Fig. S3). The number of IBD blocks for the cultivars in the Ningyou7 pedigree was greater than that in the Tapidor pedigree (Fig. 4), which provides evidence of a greater power of selection or increased recombination occurring in the recent breeding of Ningyou7^26–28^. The IBD regions that Regent inherited from the two grandparents studied here summed to only about 176 Mb (Table 1), only a subset of the 650 Mb covered by the SNP markers (Fig. 4). This can be attributed to Regent having inherited almost 75% of its genome complement from Turret^5^. A similar consequence of complex or unbalanced pedigree construction appears to account for the relatively low level of 202 Mb IBD regions for Tapidor derived from the parents and grandparents studied here (Fig. 4; Table 1). In this case, Tapidor has derived part of its genome complement from SI8879 as well as from Turret^7^ (Fig. 1). Overall, the total IBD length in the C subgenome was larger than that in the A subgenome in both pedigrees (Fig. 4), suggesting that the C subgenome of parental cultivars has contributed more to the selected agronomic traits of current cultivars than the A sub-genome, whether bred in Europe or in China. The relative conservation of the C subgenome during breeding of both Tapidor and Ningyou7 is consistent with observations that i) canola OSR cultivars show less genetic variation in the C compared with A subgenomes^11^ and ii) LD decay of canola OSR cultivars is much larger in the C than in the A subgenome^37^.

The QTLs for flowering time, seed oil content, seed glucosinolate content, seed erucic acid content, seed protein content and root morphological traits were reanalyzed using the new high-density SNP-based *Bna*TNDH linkage map^36^ (Luo *et al*. unpublished data). The QTLs and putative genes located in the IBD regions of cultivars within the Tapidor and Ningyou7 pedigrees illustrate the differences in haplotype transfer from the progenitor cultivars to Tapidor and Ningyou7 (Fig. 6 and Supplementary Table S2). The 18 favorable gene clusters identified in the Ningyou7 pedigree were involved in six important agronomic traits, while the two favorable gene clusters identified in the Tapidor pedigree were only associated with seed glucosinolate and protein content (Fig. 7 and Supplementary Table S2). Half of the 24 favorable gene clusters were associated with two or more important agronomic traits, suggesting that agronomic traits such as flowering time and root morphology could influence seed quality traits, including oil, erucic acid, protein, and glucosinolate content^14, 23, 38^. This might be due to pleiotropic effects when a favourable gene substitution occurs in a population, or due to changes in gene frequencies at closely linked loci^39^.

Genes with lower gene diversity in a population tend to reflect selection in the breeding of cultivars^29, 40^. In our study *BnaA05g03310D* (BnaA.FPA), which is the *B. napus* ortholog of the flowering time gene *AT2G43410* in *A. thaliana* and *Bra004761* in *B. rapa* (Chiifu-401), was detected within the IBD block on A05 with lower gene diversity in two early breeding cycles in pairwise comparison of progenitor cultivars Chunayou2 *vs* Shengliyoucai, Ningyou7 *vs* Shengliyoucai and Ningyou7 *vs* Chuanyou2 from the Ningyou7 pedigree (Fig. 7). The donor parent of *BnaFPA* is Shengliyoucai and the gene was transferred via Chuanyou2 to Ningyou7. In south China, early flowering results in early harvest of OSR, which can alleviate loss of seed yield due to high temperatures from late April to early May.

Plant roots are of fundamental importance to soil resource acquisition^41^. Phosphorus acquisition can be improved by increasing the lateral roots distributed in the topsoil^42^. An ortholog of *AtIAA28* was found within an IBD block on A06 which is conserved from Shengliyoucai to Chuangyou2 and hence to Ningyou7, and is co-located within a QTL for lateral root development (Fig. 6 and Supplementary Table S2). Genes homologous to *AtPht1;9* are found within IBD blocks on A09 conserved from Shengliyoucai to Chuanyou2 in the same pedigree, and are collocated within a QTL for primary root length and seed yield per plants at both low and high phosphorus supply (Fig. 6 and Supplementary Table S2). In the Tapidor pedigree, orthologous alleles of *AtPht1;9* were conserved from Bienvenu to Tapidor. Genes that have been subject to breeding selection show lower gene diversity (Supplementary Table S2). The *B. napus* orthologue of *A. thaliana AtPht1;9*, *BnaA03g35470D*, is located on chromosome A03 and has also been detected as a contributor to responses to phosphorus deficiency in a *B. napus* population of 405 inbred lines (Wang *et al*., unpublished data). Phosphate fertilizers were used extensively in European agriculture^43^ during the period Tapidor was bred from Liho and Bronowski, whilst little phosphate fertilizer was being used in China during the time Ningyou7 was being developed from Shengliyoucai and Chengduai. It is possible, therefore, that differences in root morphology between Tapidor and Ningyou7^23, 36^ might be attributed to selective breeding under these contrasting conditions.

Although the *Brassica* 60 K Infinium SNP array^44^ provides a straightforward and powerful platform to facilitate assessment of genetic variation among *B. napus* cultivars, we recognize that the detection of SNP variation may be biased due to the relatively small number of genotypes that were originally used to identify SNPs. Since sequence data from both Ningyou7 and Tapidor as samples in the development of the SNP array^45^, this could affect the utility of Tajima’s D as a measure of the evolutionary history of a locus. In particular, this could occur since Tajima’s D is based on detection of pairwise differences between samples. An example of this potential bias is apparent in the detection of only one *FAE* (*KCS18*) locus on A08, whereas the corresponding C genome copy on C03 was not detected as a candidate gene, although it had previously been identified in contributing to the low erucic acid phenotype of Tapidor^19^. Moreover, the detection of candidate genes and estimates of gene diversity based on array data does not take into account those polymorphisms that may be present between Tapidor and Ningyou7 but not represented on the array. Based on the transcriptome data of Bancroft *et al*. (2011)^1^, 19 of 105 candidate genes within IBD blocks and QTL had between 1 and 12 SNPs between Tapidor and Ningyou7 and appear to be associated with the set of traits investigated in our study (Supplementary Table S3). Unfortunately we were not able to differentiate gene homologs as the transcriptome data are not yet anchored to physical map positions^1^. However, the polymorphisms that were discovered could be used to generate molecular markers to assign gene loci to specific QTL regions.

The total IBD block length in the C subgenome was larger than that in the A subgenome in both the Tapidor and Ningyou7 pedigrees, suggesting that the C subgenome had probably contributed more to agronomically useful traits than the A subgenome in the breeding of OSR in both Europe and China. The genetic differences in improved traits (flowering time; seed yield; seed oil, glucosinolate and erucic acid content; and root traits for P uptake) during the breeding history of Tapidor and Ningyou7 have been identified from IBD, QTLs and candidate genes, as well as with Tajima’s D and a gene diversity test in the two pedigrees. Twenty four favorable gene clusters were detected as being transferred from the progenitor parents to Tapidor and Ningyou7, and could be used for further improving and dissecting the agronomic traits of *B. napus*.

## Methods

### Plant materials

Tapidor pedigree cultivars originate from European countries and Canada. Among these Liho came originally from Germany, Bronowski from Poland, Bienvenu and Tapidor from France, and Regent from Canada. Ningyou7 pedigree cultivars originate from Asian countries, with Shengliyoucai from Japan, and Chengduai, Chuanyou2, Ningyou1 and Ningyou7 from China. The species and geographical origin and basic traits of all cultivars studied are summarized in Supplementary Table S4, and their pedigrees are summarized in Fig. 5. Seeds were provided by Jinling Meng and grown in the field in Wuhan (114°20′54″E, 30°28′36″N) with self-pollination. Traits (seed oil content; seed glucosinolate content; seed erucic acid content; seed protein content) were assessed by near-infrared reflectance spectroscopy (NIR; Systems 5000)^11^ and flowering time assessed according to Long *et al*.^17^. Root traits and phosphorus uptake were evaluated according to Shi *et al*. and Zhang *et al*.^23, 36^ (Supplementary Table S5).

### Collection of agronomic traits and QTL mapping

#### Data collection

Data for flowering time of the *Bna*TNDH population and its parental lines, Tapidor and Ningyou7, were mostly obtained from previous studies^17^ across 11 environments over four years. Flowering time was calculated as the time from sowing to the date when the first flower of half of the plants in the plot had opened. Lines that did not flower in all the spring-cropped environments were given a score of 150 days^18, 46^. Data for seed oil, glucosinolate, erucic acid and protein content of the *Bna*TNDH population and its two parents were also acquired primarily from previous studies^13, 14^ across 15 environments over five years. In these studies approximately 3 g seed per accession were analysed using NIRS on a reflectance scanning mode^14^. Root morphology data for the *Bna*TNDH population and two parents grown at low phosphorus (LP) and high phosphorus (HP) supply were obtained from a previous study by Shi *et al*.^23^. The plants were grown at LP (0.006 mM P) and HP (0.625 mM P) supply on agar, with ample supply of all other nutrients. The trays were scanned at 12 d. Seedling traits, including primary root length, lateral root number, lateral root density, lateral root length, root and shoot fresh weight, were determined.

#### QTL mapping and prediction of candidate genes underlying the QTL regions

All the data for flowering time, seed quality and root morphology traits from the *Bna*TNDH population were used to conduct QTL analysis using the *Bna*TNDH 2041 linkage map, a new high-density SNP-based genetic linkage map^36^ (Luo *et al*., unpublished data). QTL detection employed the composite interval method (CIM) using QTL cartographer software version WinQTLCart2.5^47^. SNP markers flanking the QTL regions were mapped on the genome of Darmor-*bz* ^4^. The genes related to the studied traits located in the homologous physical regions of Darmor-*bzh* were predicted to be the candidate genes.

### Genotyping

#### DNA extraction

A modified cetyltrimethyl ammonium bromide (CTAB) method was used to extract DNA from young leaves^48^. Four leaves from different individuals of each line were used to construct DNA bulks. The DNA concentration in bulks was measured by electrophoresis through a conventional 2% agarose gel. 30 ng μl^−1^, 50 ng μl^−1^ and 100 ng μl^−1^ λDNA (48502 bp) was used as reference. The final DNA concentration was adjusted to be 50 ng μl^−1^.

#### Microarray hybridization and SNPs identification

The *Brassica* 60 K Illumina Infinium SNP array (Illumina, USA) contains 52,157 probes, each representing a distinct locus. Microarray hybridization consisted of the following steps: I, The DNA samples were denatured and neutralized to be prepared for amplification; II, The amplified product was fragmented by MSM enzyme; III, After precipitation using isopropanol, the fragmented DNA was collected by centrifugation at 4 °C and the precipitated DNA was re-suspended and in hybridization buffer at 48 °C and denatured at 95 °C; IV, Re-suspended DNA were added to a beadchip for hybridization at 48 °C; V, Using the captured DNA as a template, oligos were extended on the beadchip by a single base; VI, The Illumina HiScan was used to scan the beadchip, using a laser to excite the fluorophore of the single-base extension product on the beads. The scanner recorded high-resolution images of the light emitted from the fluorophores, which were analysed using the Illumina HiSeq 2000. The fluorophores had red and green colors, which represented four different types of SNP marker (A, T, C and G). Genetic loci were identified by statistical analysis (*Infinium* HD Assay Ultra, Manual, Experienced User Card)^14, 18, 35^.

### Physical mapping of SNP markers

The *Brassica* 60 K Illumina Infinium SNP array was designed based on reference sequences of *B. rapa* (AA) and *B. oleracea* (CC)^14^. Each of the SNP markers is attached to a length of 50 bp SNP probe. The genome of Darmor-*bzh* was used as the reference genome and all the SNP markers were mapped to its physical map. Only one copy of BLAST hits of the SNP marker against the reference sequences were considered to be the most likely SNP position^14^.

### Identity by descent (IBD) analysis in Tapidor and Ningyou7 pedigrees

Identity by descent (IBD) is a segment on the chromosome representing a haplotype block inherited from a shared common ancestor^24^. The software fastIBD (Beagle version 4.0)^49^ was used to detect IBD between pairs of cultivars among the Tapidor and Ningyou7 pedigrees using genome-wide SNP data. The software takes account of haplotype frequencies and uncertain haplotypes whilst enabling fast computation using genome-wide SNP data. Linked markers were identified at a P value(E) < 10^−9^.

The distributions of IBD regions in Tapidor and Ningyou7 pedigrees were represented using R3.1.3 plotting software^3^. Using SNP markers for Tapidor or Ningyou7 as a reference, IBD regions for pairs of cultivars within the respective pedigrees were mapped. Based on the physical map (anchored genome sequence) of Darmor-*bzh*, the genes detected both within the QTL regions detected in the *Bna*TNDH population, and within the IBD region of the progenitors of Tapidor and Ningyou7, were used to identify gene clusters.

### Tajima’s D test and Gene diversity

Tajima’s D test is used to determine where selection has occurred at the DNA level based on the neutral selection theory of populations^29^. The Dnasp5 software^50^ uses a sliding window method (segment of DNA) which progressively scans along the genome sequences in steps, and was employed to calculate parameters across a DNA region. The parameter is calculated in each window, and the value assigned to the nucleotide at the midpoint of the window. The D pair statistic is an estimate of the diversity level of the five cultivars within each pedigree using a sliding windows analysis, and is able to establish the variation of a unit of 15 loci with a single locus at each step^29^.

PowerMarker V3.25 was employed to calculate allelic frequency differences, in order to speculate on the genetic difference due to mutations and selection^29, 51^. The genome wide distribution of gene diversity for each of the 19 chromosomes was assessed in the *Bna*TNDH population. The selection loci in genomes of the Tapidor and Ningyou7 pedigrees were determined by combining the gene diversity test and the candidate genes underlying the QTL regions. A higher frequency of selection events is associated with regions with lower gene diversity. Based on two main breeding selection cycles, the gene diversity changes among three cultivars were calculated. These cultivars were earlier breeding cycle cultivars (Liho, Bronowski and Regent) and later breeding cycle cultivars (Bienvenu, Regent and Tapidor).

Nei’s measure of the average gene diversity per locus H_S_ is determined by the formula^30^:

$${H}_{s}=\frac{1}{k}\cdot \sum _{s=1}^{k}(H{s}_{s})=\frac{1}{k}\cdot \sum _{s=1}^{k}[1\,-\,{{q}_{s}}^{2}-{(1-{q}_{s})}^{2}]$$

where k is the total number of loci (differentiating factors), *H*_*S*s_ = 1 − *q*_s_^2^ − (1 − *q*_s_)^2^, and q_s_ is the frequency of one of the two alleles at the *s*th diallelic locus (or virulence frequency, or band frequency, or frequency of appearance 1 at the *s*th differentiating factor).

### Linkage disequilibrium (LD)

The model of non-linear regression in TASSEL 3.0^14^ was used to calculate the LD of whole-genome of the ten cultivars in Tapidor and Ningyou7 pedigrees. The full matrix LD model was chosen and the correlation coefficient (r^2^) for pairs of markers was calculated using a sliding window with increments of half a window each step^52^. A curved line was fitted based on X (r^2^) and Y (physical distance) and r^2^ was 1 while physical distance of two loci equal to 0 bp. When r^2^ fell below 0.2, the physical distance was estimated^53^. Where genes are located within a region of linkage disequilibrium (LD ≤ 0.7 Mb) in the *B. napus* genome they will form a cluster, and are more likely to be transferred as an intact haplotype block from the parents to offspring.

## Electronic supplementary material

Supplementary Dataset 1

Supplementary Dataset 2

Supplementary Dataset 3

Supplementary information
