# Supplementary material for: Breeding histories and selection criteria for oilseed rape in Europe and China identified by genome wide pedigree dissection
Source: Sci Rep. 2017 May 15;7:1916. doi: 10.1038/s41598-017-02188-z (PMC5432491; doi:10.1038/s41598-017-02188-z)
Supplement: Supplementary file 4 — Supplementary information [file 41598_2017_2188_MOESM4_ESM.docx]

**Breeding histories and selection criteria for oilseed rape in Europe and China identified by genome wide pedigree dissection**

Xiaohua Wang^1,2^, Yan Long^1,3^, Nian Wang^4^, Jun Zou^1^, Guangda Ding^1,2^, Martin R. Broadley^5^, Philip J. White^6,7^, Pan Yuan^1,2^, Qianwen Zhang^1,2^, Ziliang Luo^1^, Peifa Liu^1^, Hua Zhao^4^, Ying Zhang^1,2^, Hongmei Cai^2^, Graham J. King^1,8^, Fangsen Xu^1,2^, Jinling Meng^1^, Lei Shi^1,2^ ⃰

**Supplementary Figures**

**Supplementary Figure 1.** Variation in Tajima’s D values each chromosome for cultivars in the Tapidor (red) and Ningyou7 (green) pedigrees. Key genes controlling flowering time (yellow stars), seed oil content (blue stars), seed glucosinolate content (orange star), seed erucic acid content (red stars), seed protein content (purple stars), and root morphological traits (pink stars) are indicated.

**Supplementary Figure 2.** Variation in gene diversity values each chromosome for cultivars in the Tapidor (red) and Ningyou7 (green) pedigrees. Key genes controlling flowering time (yellow stars), seed oil content (blue stars), seed glucosinolate content (orange star), seed erucic acid content (red stars), seed protein content (purple stars), and root morphological traits (pink stars) are indicated.

**a**

**b**

**Supplementary Figure** **3.** The transfer of identity by descent (IBD) from earlier cultivars to the Tapidor and Ningyou7 cultivars based on SNP markers. Progenitor genomes are indicated in shades of up for Tapidor (a) and shades of bottom for Ningyou7 (b). IBD of Tapidor and Ningyou7 pedigrees are showed to the right each chromosome.

**Supplementary Tables**

**Supplementary Table 1.** The SNP markers of the 60K Brassica Infinium® SNP array and the markers mapped on the genome of *cv.* darmor-*bzh* (*Brassica napus L.*)

(Excel spreadsheet)

**Supplementary Table 2.** Genes located in the QTL regions and IBD regions, and the values for Tajima's D, gene diversity and PIC (Polymorphism information content) of genes detected in genome of cultivars in Tapidor and Ningyou7 pedigrees

(Excel spreadsheet)

**Supplementary Table 3.** Alleles of candidate genes underlying IBD and QTL and gene expression in leaves of Tapidor and Ningyou7 21d after sowing

(Excel spreadsheet)

| **Supplementary Table 4.** Species and geographical origin of Tapidor, Ningyou7 and their ancestors | | | | |
| --- | --- | --- | --- | --- |
| Cultivars | Species | Originated Country | Pedigree | References |
| Liho | *Brassica napus* | German | Tapidor | Sharpe, A.G. & Lydiate, D.J. 2003; Fu, Y.B. & Gugel, R.K. 2010 |
| Bronowski | *Brassica napus* | Poland | Tapidor | Sharpe, A.G. & Lydiate, D.J. 2003 |
| Bienvenu | *Brassica napus* | France | Tapidor | Sharpe, A.G. & Lydiate, D.J. 2003 |
| Regent | *Brassica napus* | Canada | Tapidor | Sharpe, A.G. & Lydiate, D.J. 2003 |
| Tapidor | *Brassica napus* | France | Tapidor | Sharpe, A.G. & Lydiate, D.J. 2003 |
| Shengliyoucai | *Brassica napus* | Japan | Ningyou7 | Liu H, 1984 |
| Chengduai | *Brassica rapa* | China | Ningyou7 | Liu H, 1984 |
| Chuanyou2 | *Brassica napus* | China | Ningyou7 | Zhang J., Qi C., Jiang X. & Pu H. 2014 |
| Ningyou1 | *Brassica napus* | China | Ningyou7 | Chen, Z., Wei Z. & Xu, Z. 1994 |
| Ningyou7 | *Brassica napus* | China | Ningyou7 | Zhang J., Qi C., Jiang X. & Pu H. 2014 |

| **Supplementary Table 5.** Seed quality and root morphology traits of *cvs.* Tapidor and Ningyou7 and their ancestors at LP and HP | | | | | | | | |
| --- | --- | --- | --- | --- | --- | --- | --- | --- |
| Cultivars | Oil content (%) | Total Glucosinolate content (μmmol/g) | Erucic acid content (%) | Protein content (%) | PRL-LP (mm) | PRL-HP (mm) | LRN-LP (N) | LRN-HP (N) |
| Liho | 42.16 | 112.17 | 1.3 | 24.04 | 135.5 | 168.4 | 10 | 11 |
| Bronoswki | 44.84 | 23.86 | 26.54 | 24.08 | 72.4 | 116.7 | 7 | 6 |
| Bienvenu | 46.21 | 148.2 | 0 | 19.77 | 99.4 | 104.2 | 10 | 11 |
| Regent | 41.18 | 118.06 | 0.37 | 25.5 | 73.9 | 125.9 | 10 | 5 |
| Tapidor | 43.86 | 35.01 | 0 | 23.29 | 100.5 | 127.2 | 9 | 7 |
| Shengliyoucai | 43.47 | 103.48 | 31.87 | 23.43 | 79.7 | 86.9 | 10 | 8 |
| Chengduai | 32.11 | 124.79 | 26.48 | 28.72 | 63.4 | 87.8 | 15 | 8 |
| Chuangyou2 | 35.78 | 136.03 | 29.85 | 28.92 | 91.3 | 88.3 | 12 | 12 |
| Ningyou1 | 40.63 | 111.16 | 34.38 | 26.89 | 106.3 | 115.6 | 13 | 9 |
| Ningyou7 | 42.5 | 76.44 | 21.17 | 23.24 | 76.8 | 112.5 | 12 | 10 |
| Note: PRL - Primary root length; LRN - lateral root number; RDW - root dry weight; SDW - shoot dry weight. LP - 0.006 mM P; HP - 0.625 mM P. Growth period: 15 d | | | | | | | | |
